# Acute Rift Valley fever virus infection induces inflammatory cytokines and cell death in ex vivo rat brain slice culture

**DOI:** 10.1099/jgv.0.001970

**Published:** 2024-03-28

**Authors:** Kaleigh A. Connors, Zachary D. Frey, Matthew J. Demers, Zachary P. Wills, Amy L. Hartman

**Affiliations:** 1 Department of Infectious Disease and Microbiology, School of Public Health, University of Pittsburgh, Pittsburgh, PA, USA; 2 Center for Vaccine Research, University of Pittsburgh, Pittsburgh, PA, USA; 3 Department of Neurobiology, University of Pittsburgh, Pittsburgh, PA, USA

**Keywords:** Rift Valley fever virus, neurotropic virus, ex vivo model, bunyavirus, brain slice culture, viral encephalitis

## Abstract

Rift Valley fever virus (RVFV) is an emerging arboviral disease with pandemic potential. While infection is often self-limiting, a subset of individuals may develop late-onset encephalitis, accounting for up to 20 % of severe cases. Importantly, individuals displaying neurologic disease have up to a 53 % case fatality rate, yet the neuropathogenesis of RVFV infection remains understudied. In this study, we evaluated whether *ex vivo* postnatal rat brain slice cultures (BSCs) could be used to evaluate RVFV infection in the central nervous system. BSCs mounted an inflammatory response after slicing, which resolved over time, and they were viable in culture for at least 12 days. Infection of rat BSCs with pathogenic RVFV strain ZH501 induced tissue damage and apoptosis over 48 h. Viral replication in BSCs reached up to 1×10^7^ p.f.u. equivalents/ml, depending on inoculation dose. Confocal immunofluorescent microscopy of cleared slices confirmed direct infection of neurons as well as activation of microglia and astrocytes. Further, RVFV-infected rat BSCs produced antiviral cytokines and chemokines, including MCP-1 and GRO/KC. This study demonstrates that rat BSCs support replication of RVFV for *ex vivo* studies of neuropathogenesis. This allows for continued and complementary investigation into RVFV infection in an *ex vivo* postnatal brain slice culture format.

## Introduction

Rift Valley fever (RVF) is an emerging arboviral disease endemic to Africa, the Middle East, and Indian Ocean with recurrent outbreaks in livestock resulting in spillover into humans [1]. RVF virus (RVFV) infection results in a self-limiting febrile illness in people, yet some individuals develop haemorrhagic fever or late-onset encephalitis [2,3]. Fatal human neurologic disease was first documented in South Africa (1974–76) and has since been reported to be present during subsequent outbreaks in up to 20 % of severe cases [4,5]. RVFV-infected patients displaying neurologic symptoms have a 53 % case fatality rate [5], and survivors of viral encephalitis may experience long-term neurologic sequalae [2,4]. Despite these clinical outcomes, there remains limited data on RVFV pathogenesis in the central nervous system (CNS).

Our lab and others have developed animal models to study RVFV encephalitis in laboratory mice, rats, and ferrets [6,9]. In rats, inhalational exposure to pathogenic RVFV (strain ZH501) results in consistent neurologic symptoms and lethality between 7–10 days [8]. Prior studies have shown the virus enters into the CNS across the cribriform plate, replicates to high titres in brain tissue, and results in neuronal necrosis, structural damage, and haemorrhage [10]. Interestingly, blood–brain barrier (BBB) breakdown is a late pathologic event [10,11]. Previous studies provide evidence that in this rat model, RVFV enters the brain prior to weakened BBB integrity [11]. Further, additional studies demonstrated that leucocyte infiltration precedes lethality; however, the abrogation of neutrophil infiltration does not improve survival [12]. In general, viral infection in the CNS could result in virus-mediated and/or immune-mediated cytopathology, as well as disruption of neurotransmitter function, potentially leading to mortality and long-term morbidities [13,14]. Added understanding of the neuropathogenesis of RVFV infection is critical to the identification of targeted therapeutics to prevent or treat severe RVF.

As a tool to model RVFV infection within the CNS, we established *ex vivo* postnatal rat brain slice cultures (BSCs) to tractably study brain infection. Organotypic or *ex vivo* BSCs have been used to characterize other arboviral infections in the CNS, such as West Nile virus and Zika virus [15,18]. BSCs maintain the cytoarchitecture, spatial relationships, and cellular connections that may mediate viral pathogenesis. Further, viral infection can be studied without the influence of the peripheral immune system. BSCs may serve to bridge technologies between *in vitro* cell culture and *in vivo* experiments for the development and screening of novel therapeutic targets and approaches.

Here, we optimize the BSC model to demonstrate that *ex vivo* postnatal rat BSCs are permissive to pathogenic RVFV infection and support viral replication over time. Viral antigen co-localizes with neurons and glia throughout infection. *Ex vivo* rat BSCs mount an innate inflammatory and antiviral response following RVFV infection, which recapitulates the responses documented *in vivo*. We observe activation of apoptotic cell death and neuroparenchymal tissue damage throughout infection. Based on these findings, *ex vivo* rat BSCs may be used to further characterize RVFV infection in neuronal cells as an additional tool to study RVFV infection within the CNS.

## Methods

### Biosafety

All experiments with Rift Valley fever Virus were conducted in the Center for Vaccine Research (CVR) and the Regional Biosafety Laboratory (RBL) at the University of Pittsburgh following the safety procedures described previously [19]. The RBL is a registered BSL-3/ABSL-3 laboratory space with the CDC and USDA.

### Virus and cells

The ZH501 strain of Rift Valley fever virus used in these experiments was generously provided by Barry Miller (CDC, Fort Collins, Colorado) and Stuart Nichol (CDC, Atlanta, Georgia) as described previously [20]. Vero E6 (CRL-1586, American Type Culture Collection) cells were used to propagate virus following standard cell-culture conditions in Dulbecco’s modified Eagle’s medium (DMEM) containing 2 % or 10 % FBS, 1 % penicillin-streptomycin (pen/strep), and 1 % l-glutamine. For quantitation, virus was measured using viral plaque assay [7].

### Animals

Time-mated Sprague-Dawley rats (Hsd:Sprague Dawley SD) used in our study were obtained from Envigo. Litters of >6 pups were obtained at postnatal day 6 for BSC isolation. The sex of animals for BSC generation was dependent on the litter. The number of slices obtained per experiment was dependent on litter size. Experiments in this manuscript comprise eight total litters.

### Brain slice cultures

**Fig. 1.**
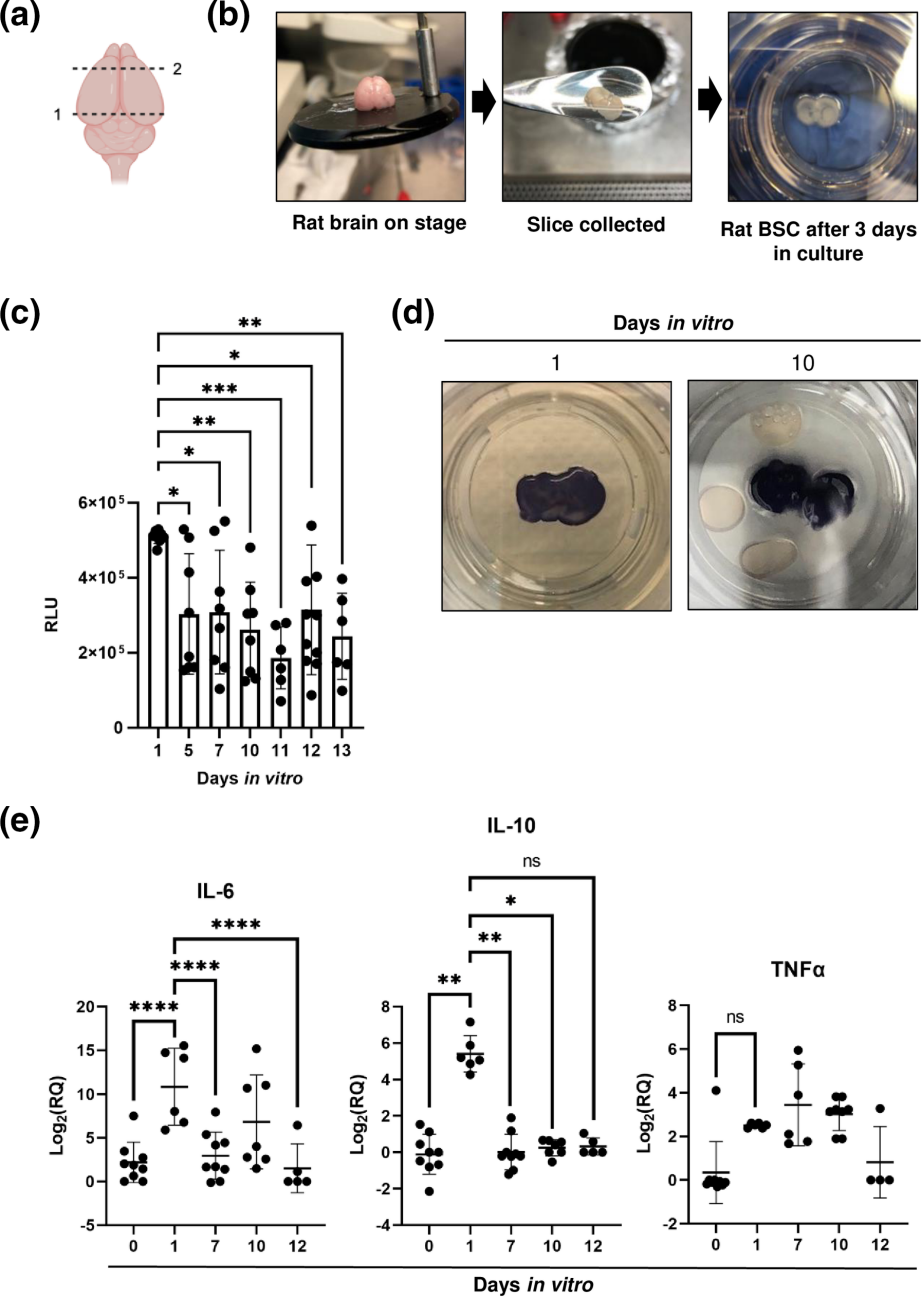
Brain slices are isolated from postnatal rats and cultured *ex vivo*. (**a**) Schematic showing two cuts to whole brain (created in BioRender). Removal of the cerebellum and brain stem (cut 1) prior to mounting onto a vibratome stage. Removal of bi-lobular frontal cortex (cut 2). (**b**) Sequential, coronal brain sections were isolated from 6-day-old rat pups using a vibratome. Then, 400 µm sections were collected and seeded on PFTE membrane inserts. (**c**) Cellular cytotoxicity over time in relative luminescence units (RLU), quantified using LDH cytotoxicity assay (*n*>6 slices across three experiments). (**d**) Metabolic activity observed using MTT staining (purple=active, white=dead) in two representative slices from day 1 and day 10. (**e**) IL-10, IL-6 and TNFα expression in brain slices (*n*>5 slices across three experiments) quantified by delta-delta Ct fold change (beta-actin) normalized to day 0 BSC. Error bars represent sem. Statistical analysis performed using two-way ANOVA. *P < 0.05; **P < 0.01; ***P < 0.001; ****P<0.0001; ns=not significant.

Prior to slicing, six-well plates were seeded with 10 % heat-inactivated FBS (Corning, no. 35–011-CV) in Neurobasal-A (Gibco, no. 10888022) (plating medium) and incubated at 37 °C, 5 % CO_2_. Six-day-old rat pups (P6) of mixed sex were anesthetized with isofluorane and decapitated. Whole brain was removed and placed in sterile petri dish. The cerebellum was cut with a straight razor to yield a flat coronal surface on the posterior aspect of the brain (Fig. 1a, cut 1), which was then affixed vertically to the vibratome stage with glue. Slices were obtained using a Leica VT100S (Deer Park, IL, USA) vibratome. Ice-cold dissection media (1X PBS, 5 mg ml^−1^ d-glucose, 1% penicillin-streptomycin) was used in the chamber, which was kept cold using ice cubes. Progressive 400 µm thick coronal slices were obtained at speed 8, frequency 9, after the bi-lobular frontal lobe was removed (Fig. 1a, cut 2). Between three–five slices were obtained per brain. Slices were transferred to a petri dish with cold-dissection media. Slices were then transferred to lie flat on a hydrophilic PTFE membrane (EMD Millipore, no. PICM03050) placed in each well of a six-well plate using a metal spatula. After 24 h at 37 °C, inserts and slices were transferred to new six-well plates containing Neurobasal-A with 5 % FBS. After 72 h, inserts and slices were transferred to new six-well plates containing Neurobasal-A with 1×B27-A (Gibco, no. 12587010). Media was changed every other day until 10 days *in vitro* (DIV) when slices were transferred to into the BSL-3 for infection with RVFV. BSCs were infected with RVFV diluted in DMEM/2 % FBS at 1×10^3^, 1×10^4^, or 1×10^5^ total p.f.u., or mock infected in DMEM/2 % FBS, by dropping 100 µl on top of the slice and placing 900 µl around the PTFE insert [21]. Slices were incubated with virus for 1 h, then the PTFE insert was transferred to a new plate containing fresh Neurobasal-A with 1×B27-A media without virus. Slices were not washed to avoid damage. Time-matched mock infected slices were used as controls and pooled unless otherwise noted.

### Viability of BSCs

Release of lactate dehydrogenase (LDH) from damaged cells was assessed using LDH Glo Cytotoxicity Assay (Promega, no. J2381). Culture media was obtained from slices starting a 1 DIV, and collected at days 5, 7 and 10 prior to infection. During infection, culture supernatant was obtained from mock infected controls to generate data from days 11, 12 and 13 DIV. MTT [3-(4,5-dimethylthiazol-2-yl)−2,5-diphenyltetrazolium bromide] (Invitrogen, no. M6494), which is reduced by viable cells to yield blue precipitate, was diluted to 0.5 mg ml^−1^ in 1X PBS. MTT was added around the PTFE insert and plates were incubated for 45 min at 37 °C and imaged using an iPhone.

### RNA extraction and quantitative RT-qPCR

Total RNA from supernatant was extracted as previously described to quantify RVFV viral RNA [19]. For cytokine/chemokine measurements, single rat BSCs were homogenized in 200 ul complete media through agitation with a pipet. Total RNA was extracted from 100 uL of BSC homogenate using the RNAeasy mini kit (Invitrogen, no. 12183025) with DNAse treatment. cDNA was synthesized using M-MLV reverse transcriptase (Invitrogen, no. 28025013) with random hexamer primers. Semi-quantitative real-time PCR was used as previously described [22] for the detection of cytokines and chemokines (IFN-α, IFN-β, IFN-γ, IL-1β, IL-18, IL-6, TNF-α, GRO/KC, MCP-1/CCL2) normalized within each slice to housekeeping gene β actin.

### Immunofluorescent staining

Slices were fixed in methanol-free 4 % paraformaldehyde for 24 h at 4 °C, removed from PTFE inserts, washed 2× in PBS and stored at 4 °C. Slices were permeabilized by incubating in a solution of 4 % FBS and 0.3 % Triton X-100 in PBS (BPS) for 45 min at RT. Slices were incubated in primary antibody diluted in BPS, either for 2 h at RT or overnight at 4 °C with rocking. Slices were washed in BPS 3× for 10 min, then incubated in secondary antibody diluted in BPS for 2 h at RT, rocking. Primary antibodies in this study include anti-rabbit RVFV NP pAb (GenScript, no. R03451, 1 : 300), anti-mouse RVFV-NP (BEI Resources, no. NR-43188, 1 : 300) anti-rabbit IBA-1 (FujiFilm, no. 019–19741, 1 : 1000), anti-chicken GFAP (ABCam, no. AB4674, 1 : 500), anti-chicken beta III tubulin (Millipore Sigma, no. AB9354, 1 : 500), anti-mouse NeuN (EMD Millipore, no. MAB377, 1 : 500). Slices were counterstained with Hoechst, washed again with BPS and transferred to glass slides with bridge mounts. Slides were cleared by adding alkaline solution and incubating 5 min at RT in the dark [23]. Cover glass was then affixed over the bridge-mounted slices. Slices were imaged on a Nikon A1 confocal at the Center for Biologic Imaging or a Leica DMI8 inverted fluorescent microscope at the Center for Vaccine Research. Images were obtained at 2.5X (Leica) or 20X (Nikon A1) magnification. RVFV-infected BSCs were treated as primary deletes and served as imaging controls. All 20X images are Maximum Intensity Projects of 40 µm z-stack. Images were processed using ImageJ.

### Statistical analysis

All statistical analyses were performed in GraphPad Prism software (La Jolla, CA). For Fig. 1b, d, 5a and 6, two-way ANOVA was used to determine statistical significance between groups at each time point (*, *P*<0.05; **, *P*<0.01; ***, *P*<0.001; ****, *P*<0.0001). Multiple comparison was performed using Tukey’s multiple comparison test.

**Fig. 2. F2:**
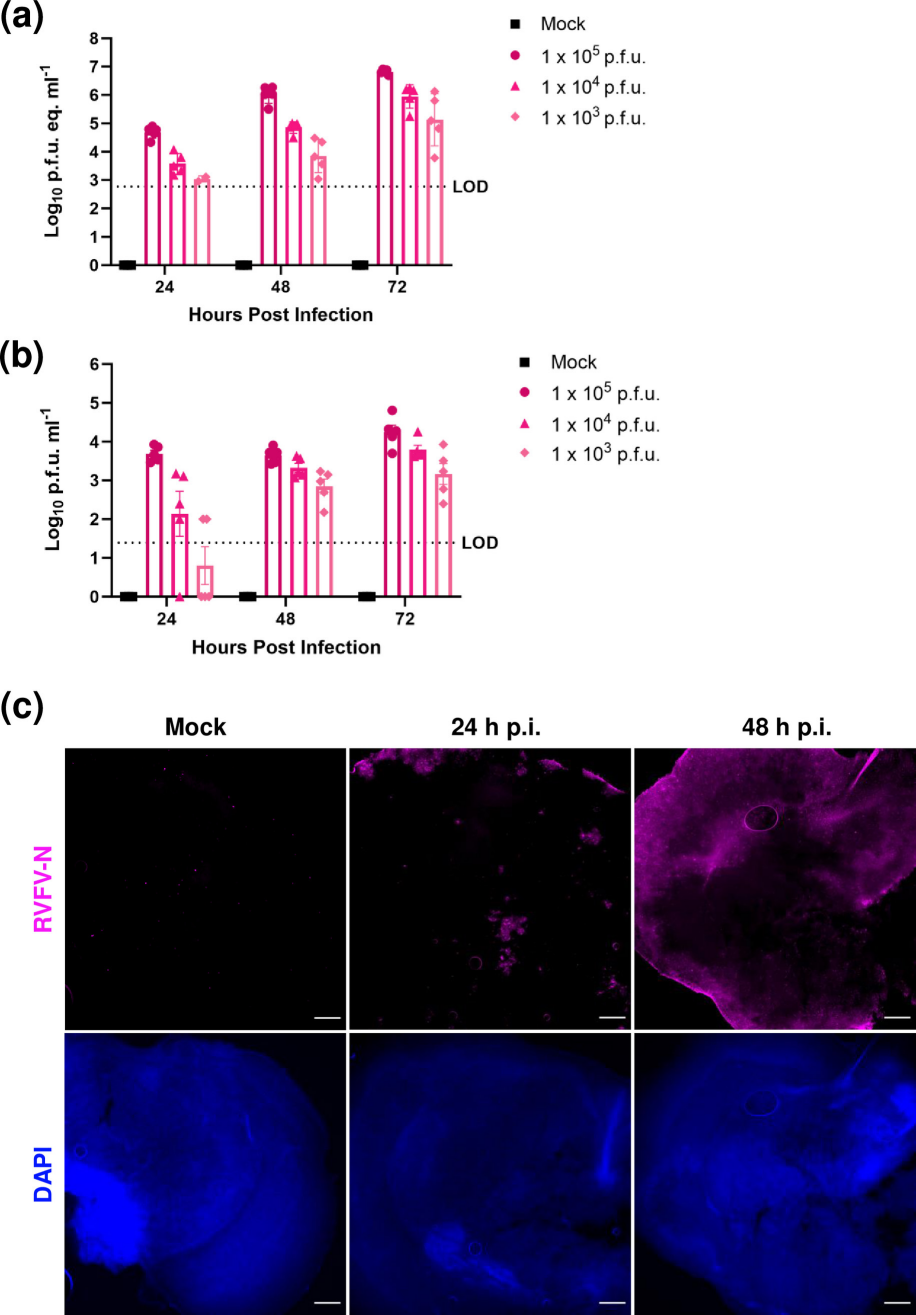
RVFV infects, replicates, and spreads in *ex vivo* rat BSCs. Rat BSCs (*n*=5 per timepoint) were inoculated with 1×10^5^, 1×10^4^ or 1×10^3^ p.f.u. of pathogenic RVFV (strain ZH501) or mock infected and viral RNA (**a**) or infectious virus (**b**) was quantified at 24, 48 and 72 h p.i. (**c**) Mock or RVFV-infected slices at 24 h p.i. or 48 h p.i. were stained with anti-RVFV-N (magenta) and counterstained with DAPI (blue). Slices were cleared and imaged at 2.5× magnification. Scale bar=100 µm. Images are representative of five slices imaged per time point.

**Fig. 3. F3:**
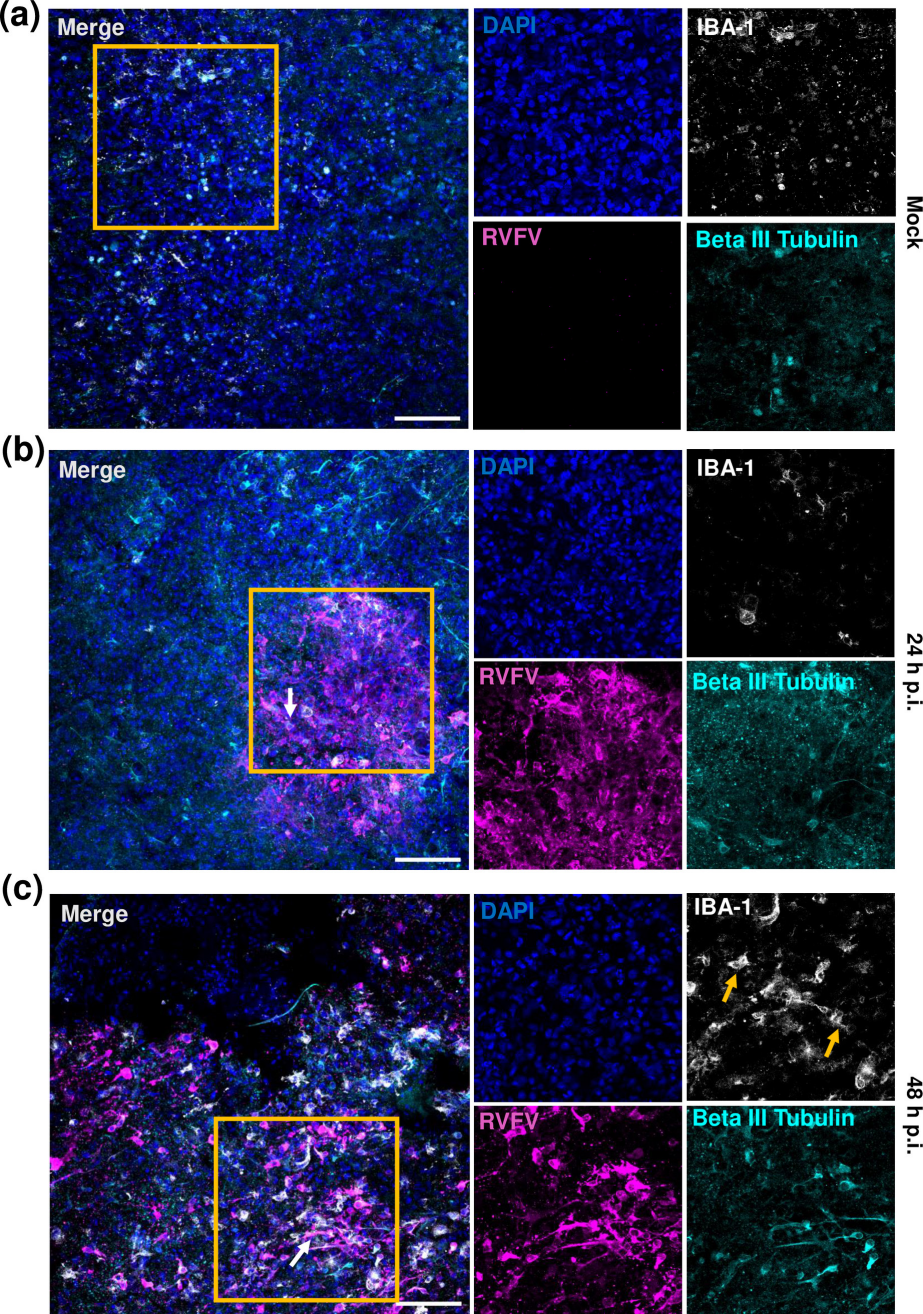
Rift Valley fever virus infects neurons and induces microglial changes in rat BSC. (**a–c**) Slices were fixed overnight with 4 % paraformaldehyde at different timepoints following infection with 1×10^5^ p.f.u. pathogenic RVFV (strain ZH501) or mock-infected control. Whole slices were stained with anti-RVFV-N (magenta), anti-IBA-1 (white), and anti-Beta III Tubulin (cyan), and counterstained with DAPI (blue). Slices were cleared, mounted to slides, and imaged at 20× on a Nikon A1 confocal across 40 µm z-plane. Yellow boxes over merged image are areas which are shown as single colour channels on the right. White arrow=co-localization of beta-III tubulin and RVFV-N. Yellow arrow=IBA-1+ microglia. Images represent maximum intensity projection. Scale bar=100 µm.

**Fig. 4.**
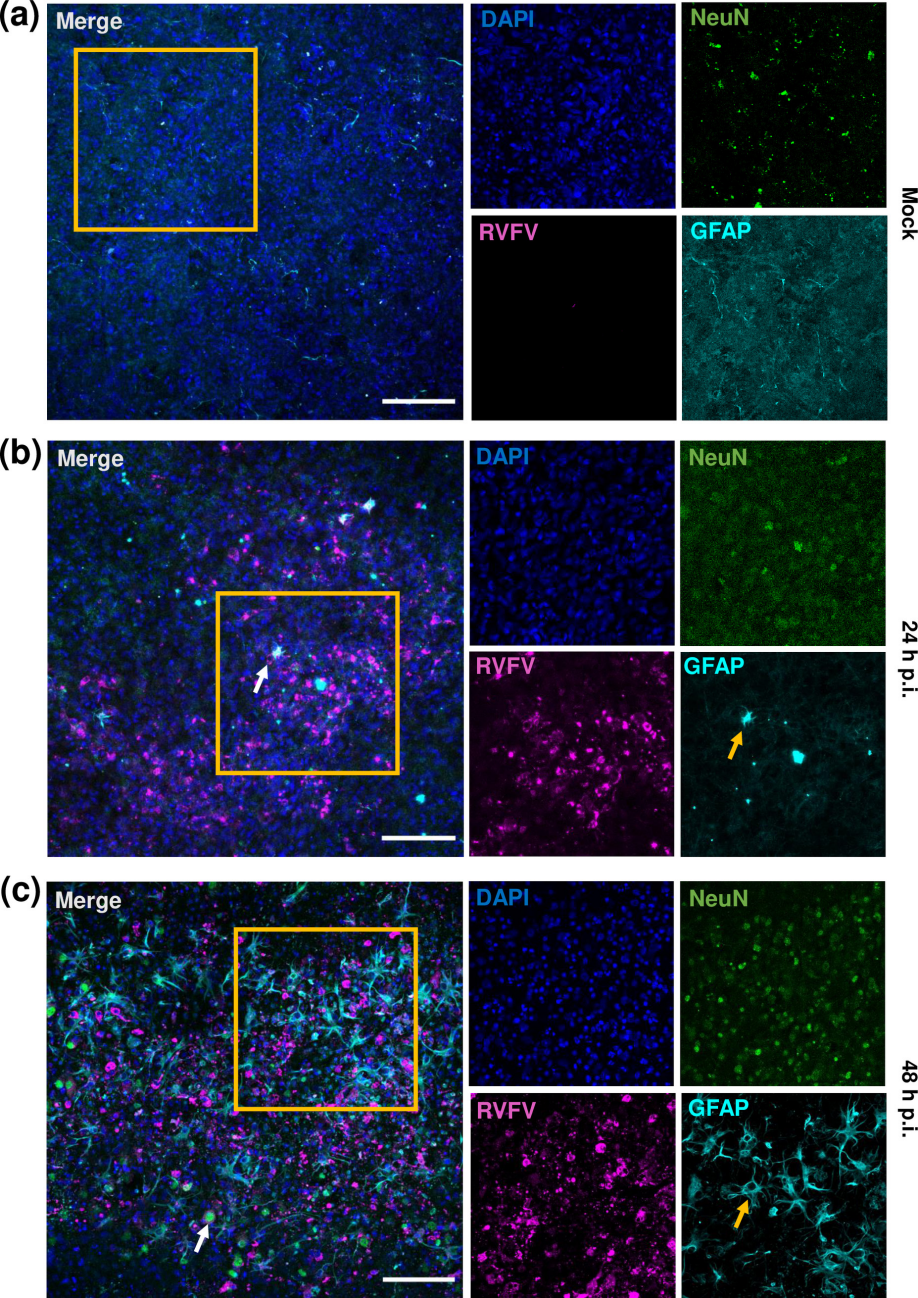
Rift Valley fever virus infection of rat BSCs promotes astrocyte activation. (**a–c**) Slices were fixed overnight with 4 % paraformaldehyde at different timepoints following infection with 1×10^5^ p.f.u. pathogenic RVFV (strain ZH501) or uninfected control. Whole slices were stained with anti-RVFV-N (magenta), anti-NeuN (green), and anti-GFAP (cyan), and counterstained with DAPI (blue). Slices were cleared, mounted to slides, and imaged at 20× on a Nikon A1 confocal across 40 µm z-plane. Yellow boxes over merged images are areas which are shown as single-colour channels on the right. White arrow=co-localization of NeuN and RVFV-N. Yellow arrow=GFAP+astrocyte. Images represent maximum intensity projection. Scale bar=100 µm.

## Results

### *Ex vivo* rat brain slices are viable in culture for at least 12 days

Previous studies using rat BSCs demonstrated an inflammatory response immediately after slicing that resolves over time, and the BSCs remain viable for a surprising amount of time in culture [24]. To characterize the BSC system for our studies, whole brains were isolated from 6-day-old rat pups. The cerebellum was removed to create a flat mounting surface (Fig. 1a, cut 1), and the brain was glued to a stage. Using the vibratome, a cut was made to remove the bi-lobular frontal lobe, which includes the olfactory bulb (Fig. 1b, cut 2), thereafter sequential 400 µm coronal sections were obtained (up to five slices per brain) (Fig. 1b). BSCs were cultured on a polytetrafluoroethylene (PTFE) membrane to allow for air–liquid interface with media changes every 2–3 days. Slices become thinner over time in culture and brains become more transparent and maintain transparency over the culture period (Fig. 1b), which is supported by previous literature [25,26]. Tissue viability over time was determined by two methods: quantification of lactate dehydrogenase (LDH) in the culture supernatant and metabolic activity over time. LDH is a stable cytoplasmic enzyme that is rapidly released into cell culture when the plasma membrane is damaged. Serial collection of supernatants from the wells of brains through 13 days *in vitro* (DIV) show a decrease in LDH release following slicing, indicating that the damage from slicing resolved over time in culture (Fig. 1c). MTT [3-(4,5-dimethylthiazol-2-yl)−2,5-diphenyltetrazolium bromide] is an indicator of viability using cellular metabolism. MTT is a yellow coloured, water-soluble tetrazolium salt that is converted in viable cells to dark purple insoluble formazan by enzymes in active mitochondria. MTT was added to the culture wells of rat BSCs for 45 min at 37 °C. Viable BSCs turn dark purple, while BSCs that are dead will remain white. Rat BSCs incubated at 1 or 10 DIV turn purple (Fig. 1d), which suggests sustained metabolic activity throughout the culture period.

It has been shown that the trauma of slicing and adjustment to culture induces an inflammatory response in BSCs [24]. As viral infection can perturb and be influenced by inflammatory responses, it was important to establish the baseline inflammatory profile of rat BSCs through culture prior to viral infection. To determine the inflammatory response following isolation and slicing, whole brains were collected at timepoints throughout culture and RNA was extracted. Using delta-delta Ct quantification of fold change relative to a 0 h slice control, we calculated changes in gene expression in rat BSCs across 12 DIV. Relative quantification of pro (IL-6 and TNF-α) and anti-inflammatory (IL-10) cytokines demonstrate rat BSCs mount an inflammatory response following slicing at 1 day that significantly decreases during culture period (Fig. 1e). Taken together, we demonstrated that BSCs maintain viability and have reduced inflammatory response following slicing at least up to 12 DIV. Because of this, we chose to infect all BSCs at 10 DIV moving forward.

### Rift Valley fever virus infects and replicates in *ex vivo* rat brain slices over 72 hours

To determine permissivity of rat BSCs to RVFV infection, we infected slices at 10 DIV with pathogenic RVFV (strain ZH501) at multiple input doses (1×10^3^–1 x 10^5^ total p.f.u.). Mock-infected slices were obtained at each timepoint to serve as uninfected controls. Quantification of viral RNA and infectious virus over time both demonstrate that rat BSCs are permissive to infection and support viral replication in a dose-dependent manner (Fig. 2). Virus replicates from 1×10^5^ to 1×10^7^ p.f.u. eq ml^–1^ by 72 h p.i. (Fig. 2a). A dose-dependent replication is observed by both RNA quantification and infectious titre, though several slices of BSCs infected with 1×10^3^ p.f.u. were below the limit of detection at 24 h p.i. (Fig. 2b). Given the robust viral replication observed in slices inoculated with 1×10^5^ p.f.u., all remaining experiments were carried out using this dose.

At 24 and 48 h p.i., slices inoculated with 1×10^5^ p.f.u. pathogenic RVFV were fixed overnight in 4 % paraformaldehyde then free-float stained for viral antigen (anti-RVFV N pAb) and counterstained with DAPI. Slices were cleared using alkaline solution (AKS) [23] and imaged at 2.5X on a Leica DMI8. At 24 h p.i. viral antigen is localized to the slice edges, with some areas within the slice positive for viral antigen. By 48 h p.i., viral antigen staining is observed throughout most of the slice. Images of the left hemisphere of the rat BSCs show increased viral antigen staining between 24 and 48 h p.i.Fig[Fig F2]. [Fig F2][Fig F2]4c(Fig. 2c). These findings demonstrate that RVFV actively replicates and spreads through the rat BSCs following infection.

### Rift Valley fever virus infects neurons and induces reactive microglia and astrocytes

Across slices from all time points (*n*=6, two experiments), images obtained from 40 µm z-stacks were generated, from which maximum intensity projections were created. We observed viral antigen co-localized with neurons throughout infection using neuron antibodies targeting NeuN or beta III tubulin (Figs.3  4, white arrows). We further observed activation of astrocytes and microglia using antibodies against GFAP or IBA-1. While a resting microglia phenotype was observed in mock infected slices, the microglia became bushy by 24 h p.i. (Fig. 3b) and ameboid by 48 h p.i. (Fig. 3c) in infected cultures (Fig. 3F[Fig F3]ig. 5cc, yellow arrows). Similarly, astrocyte activation was observed throughout infection with increased GFAP staining by 24 h p.i. (Fig. 4b, yellow arrows), and a more reactive astrocyte phenotype with prominent stellate processes by 48 h p.i. (Fig. 4c, yellow arrows). These data suggests that both microglia and astrocytes are activated following RVFV infection in rat BSCs.

### RVFV infection results in tissue damage and activation of apoptosis in rat BSCs

**Fig. 5.**
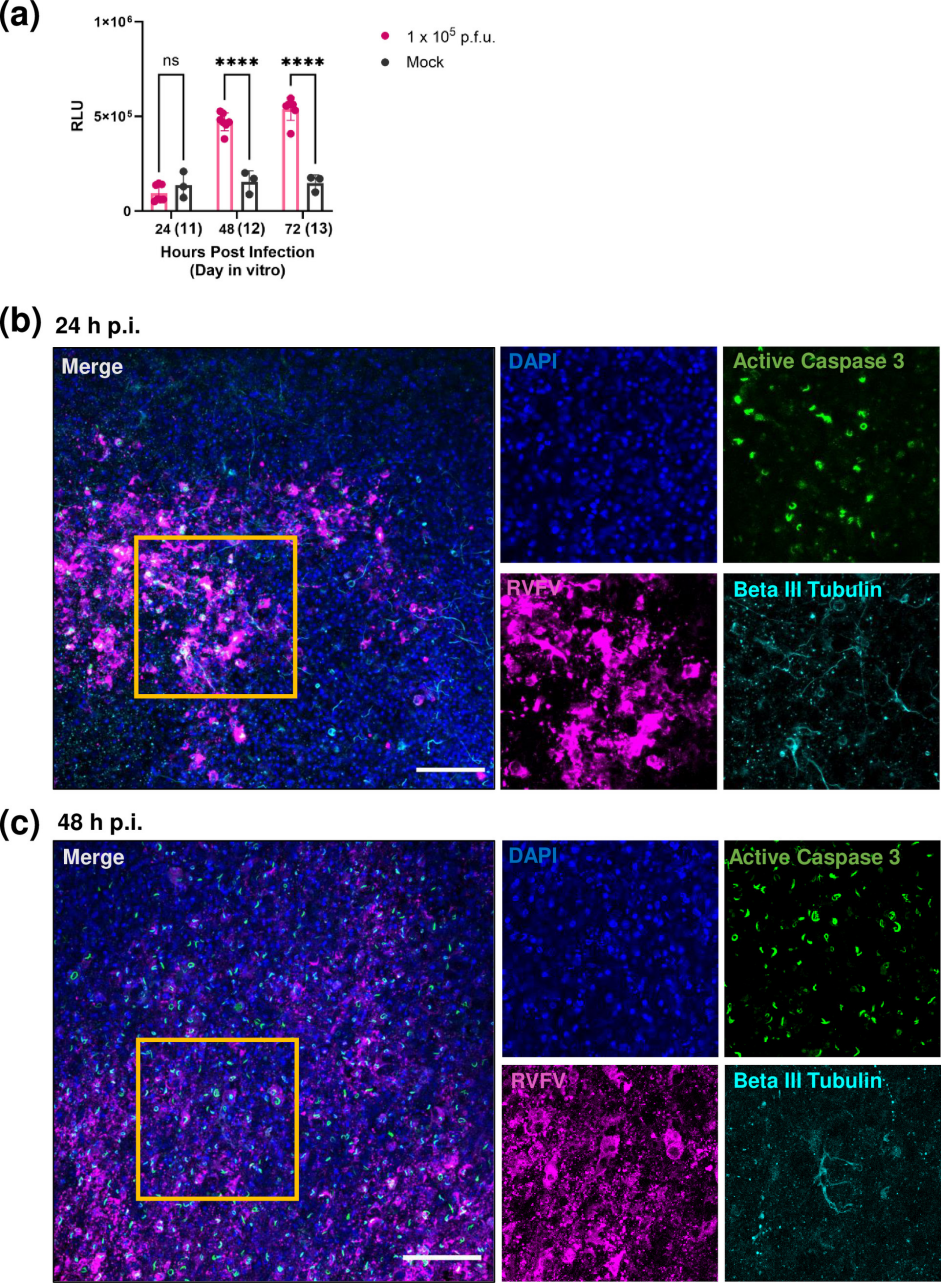
Rift Valley fever virus infection induces tissue damage and the activation of apoptosis in ex vivo rat BSC. (**a**) Cellular cytotoxicity over time in relative luminescence units (RLU) between mock and slices infected with 1×10^5^ p.f.u. pathogenic RVFV (strain ZH501), quantified using LDH Glo Cytotoxicity Assay (*n*=5 infected slices and three mock slices per timepoint). Error bars represent sem. Statistical analysis performed using two-way ANOVA. *P < 0.05; **P < 0.01; ***P < 0.001; ****P<0.0001; ns=not significant. (**b, c**) Slices were fixed overnight with 4% paraformaldehyde at 24 and 48 h p.i. following infection with 1×10^5^ p.f.u. pathogenic RVFV (strain ZH501). Whole slices were stained with anti-RVFV-N (magenta), anti-Active Caspase 3 (green), and anti-Beta III tubulin (cyan), and counterstained with DAPI (blue). Slices were cleared and mounted to slides, then imaged at 20× on a Nikon A1 confocal across 40 µm z-plane. Yellow boxes over merged image are areas which are shown as single colour channels on the right. Images represent maximum intensity projection. Scale bar=100 µm.

As RVFV replicates in the brain *in vivo* in rodent models, it results in histopathological changes including haemorrhage and structural damage [10]. To determine if RVFV-infection of rat BSCs results in tissue damage within the BSC, cell viability in supernatants from infected and mock-infected slices were compared by measuring LDH as described above. Indeed, we found significantly elevated LDH release at 48 and 72 h p.i. compared to mock-infected control rat BSCs (Fig. 5a). This suggests that RVFV infection of rat BSCs induces tissue cytotoxicity.

RVFV nonstructural protein NSs forms nuclear filaments [27], which are required for pathogenesis [28], and it was recently shown that NSs interacts with active caspase 3 within these filaments [29,30]. Using immunofluorescent microscopy, we stained infected and mock-infected rat BSCs with anti-RVFV N, anti-beta III tubulin, and anti-active caspase 3 antibodies. We observed active caspase 3 staining as filamentous structures in the nucleus of RVFV infected cells at 24 h p.i., which increased throughout the slice by 48 h p.i. (Fig. 5c). Some RVFV +cells that stained positive for active caspase 3 were also positive for neuron marker beta III tubulin. This suggests that following RVFV infection, apoptosis was induced in neuronal and non-neuronal cells within rat BSC, and active caspase 3 localized in the nucleus in RVFV-infected cells in filamentous-like structures.

### Antiviral and inflammatory response in RVFV-infected rat BSCs

**Fig. 6.**
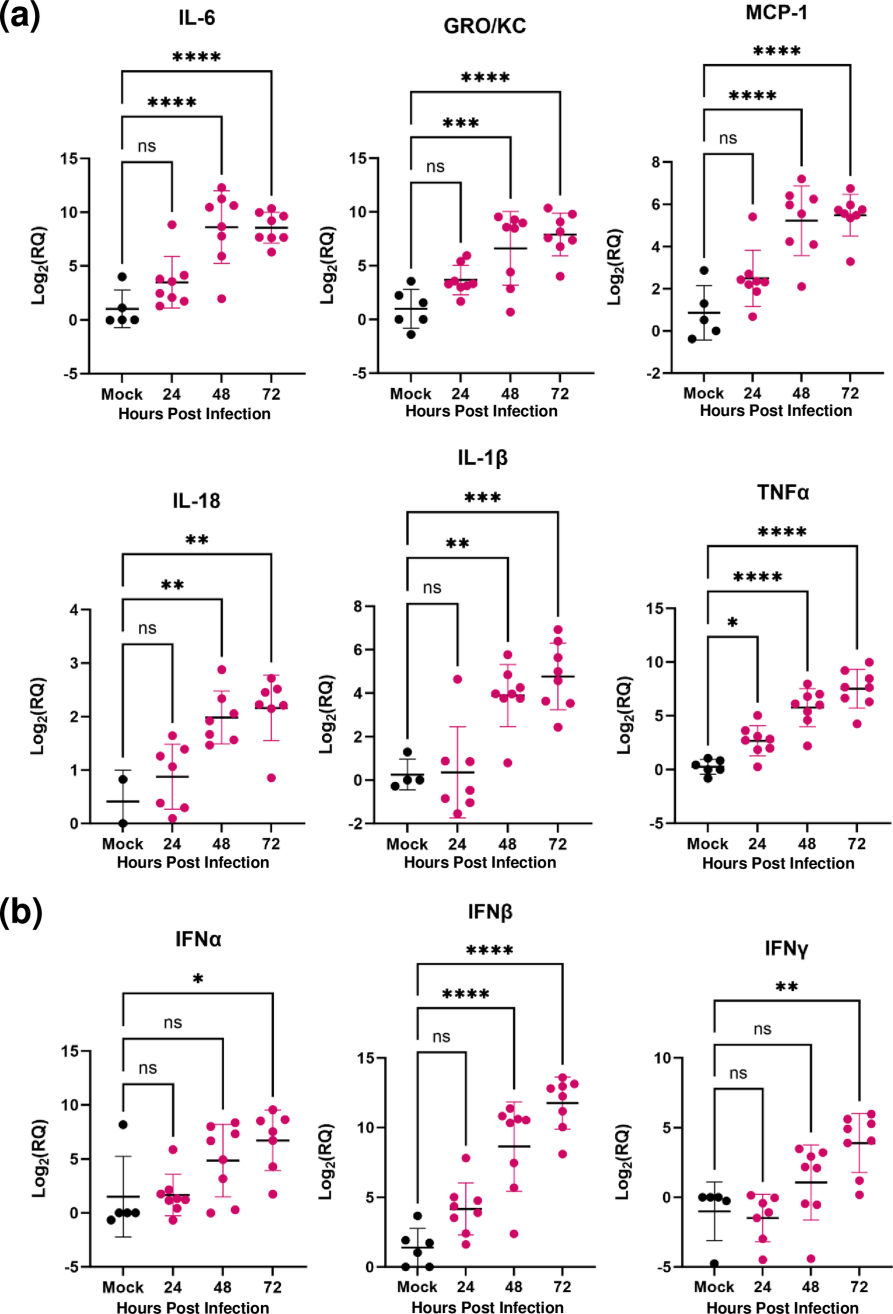
Antiviral cytokines and chemokines are expressed during RVFV infection of *ex vivo* rat brain slices. (**a, b**)Rat BSC were infected with 1×10^5^ p.f.u. pathogenic RVFV (strain ZH501) or mock infected and RNA was isolated at 24, 48 or 72 h p.i. from the whole slice. Mock data represents mock-infected slices from each timepoint, and gene-expression data was pooled. Gene expression was quantified by RT-qPCR using delta-delta Ct and normalized to the variation of the amount of beta-actin within each slice. Data collected from three independent experiments. Fold changes are relative to a 0 h mock-infected control slice. Error bars represent sem. Statistical analysis performed using two-way ANOVA. *P < 0.05; **P < 0.01; ***P < 0.001; ****P<0.0001; ns=not significant.

RVFV infection induces pro-inflammatory cytokines in the periphery and in the CNS following lethal aerosol exposure to pathogenic RVFV in rats [7,12]. In this animal model, cytokine dysregulation in the CNS precedes lethality. To determine if the cytokine response in rat BSCs reflects what is observed *in vivo*, the expression levels of a panel of inflammatory cytokines and chemokines were assessed using multiplex RT-qPCR. Infection of rat BSCs with 1×10^5^ p.f.u. of pathogenic RVFV induced expression of pro-inflammatory cytokines and chemokines IL-6, TNF-α, GRO/KC, IL-1β, IL-18, and MCP-1compared to pooled mock-infected controls. All cytokines and chemokines were significantly increased by 48 h p.i. and sustained through 72 h p.i. (Fig. 6a) . Similarly, both type I and type II interferons were induced in rat BSCs following RVFV infection. IFN-β was significantly upregulated by 48 h p.i., while both IFN-α and IFN-γ expression increased over 72 h p.i. (Fig. 6b) . These data demonstrate that rat BSCs mount an antiviral immune response following infection with pathogenic RVFV, and that the cytokine/chemokine responses in rat BSCs mirror what we have previously found in rat brains during *in vivo* infection.

## Discussion

Due to the propensity of mice to develop uniformly lethal hepatic disease after RVFV infection, understanding the neuropathogenesis of RVFV using rodents has been primarily restricted to rat studies [7,10,12]. Rodent studies using a BSL-3 pathogen such as RVFV are low-throughput and cumbersome, especially if the goal is to study complex interactions in the CNS at multiple time points after infection. On the other end of the spectrum, immortalized neural cell lines do not fully recapitulate cellular processes *in vitro*, including neuronal maturation and cellular interactions [31]. Primary neural cells, including primary neurons and primary glial cells, can be difficult to maintain in culture, although they are more representative of the capabilities and maintain similar gene expression markers compared to their counterparts *in vivo* [31].

Organotypic brain slice cultures offer an attractive intermediate and complementary model to study detailed viral–CNS interactions in a more tractable manner [32]. Cells in BSC maintain cytoarchitecture, neural connections, neural cell types and functionality found *in vivo* much more closely than cells isolated in primary culture [24]. Isolation of BSCs from three to nine day old rodents demonstrate a high degree of plasticity following the sectioning procedure and allow BSCs to be maintained for extended culture periods [33]. However, while cells within *ex vivo* BSCs undergo continued maturation and differentiation during culture, they differ from the typical maturation observed *in vivo* [24,33]. Despite these limitations, BSCs have been used with other viral systems to understand viral targets and kinetics, to assess the role of various neuronal populations in viral infection, and as a tool to screen for therapeutic efficacy [15,17, 21]. Here, we optimize the rat BSC model to study RVFV infection.

Isolation and maintenance of *ex vivo* BSCs are a well-described but technically difficult process. Using the method documented here, BSC isolated from postnatal day 6 rat pups maintain viability over time in culture based on visual confirmation of slice integrity, quantification of tissue damage, and metabolic activity. Over time, viable slices become translucent and remain so, while dead tissue turns white and becomes opaque [25]. We used LDH Glo Cytotoxicity Assay as a measurement of viability because this assay allowed individual slices to be longitudinally sampled across 13 days. Initial tissue disruption, as measured by high LDH levels at 1 DIV, was followed by a decrease in LDH release through at least 13 DIV across multiple experiments. This is similar to what has been observed elsewhere, where postnatal mouse brain slices showed high levels of propidium iodide staining at 1 DIV, which resolved over 14 DIV [34]. Viability of BSCs was further confirmed by using MTT to assess metabolic activity: viable slices stain dark purple, while slices or sections within a slice that are non-viable remain white [32]. A recent study showed that inflammatory cytokines IL-6 and TNFα were produced immediately after the trauma of slicing [24]. Indeed, in our studies, levels of IL-6, TNFα and IL-10 were elevated at 1 DIV but returned to baseline thereafter. Taken together, these data suggest that using the method presented here, rat BSCs recover and retain metabolic activity for at least 13 days after being isolated, sliced, and cultured. Thus, we determined an optimal timepoint for infection of BSCs to be at 10 DIV.

*Ex vivo* rat BSC demonstrated a dose-dependent permissivity to RVFV infection and replication. We infected the rat BSCs with multiple input doses of RVFV to assess replication kinetics over time, and this approach was based on previous studies of viral infection of *ex vivo* BSC [15,21, 35]. To ensure that we could standardize RVFV infection across potential variations within slices, we used the highest dose throughout the study (1×10^5^ p.f.u.). RVFV actively replicated and spread throughout the BSCs, and slices inoculated with the highest dose lost tissue integrity following 3 days of infection. Future studies using lower inoculation doses may allow for analysis of infected BSCs for longer times in culture.

Previous literature suggest that RVFV readily infects neurons, microglia, and astrocytes *in vitro* and *in vivo*, resulting in a highly inflammatory environment including cell death and cytokine dysregulation [6,7, 10, 12, 29, 36]. In particular, GRO/KC, IL-1β and MCP-1 are produced at high levels at end-stage disease in the brain of rats infected with RVFV by inhalational exposure [12]. *In vitro*, microglia express activation markers and produce high levels of pro-inflammatory cytokines including IFNα, IFNβ, IL-6, TNFα and MCP-1 following RVFV infection [36]. In the present study, GRO/KC, IL-1β and MCP-1 were highly expressed in RVFV-infected BSCs compared to uninfected controls. We also show morphologic changes associated with activation of microglia and astrocytes, and these cells may be the source of the detected inflammatory cytokines.

Simultaneous to viral replication and an inflammatory cytokine response, tissue damage occurred in slices infected with RVFV, as measured by LDH release, compared to uninfected slices. We stained BSCs for active caspase 3, a marker of apoptosis and found positive staining in filamentous structures within the nuclei of RVFV-infected cells. It has recently been shown that caspase 3 is activated and translocated to the nucleus where it co-localizes with the nonstructural protein NSs [29]. The rat BSC model offers an additional tool to study the mechanism of this viral–host interaction.

Use of *ex vivo* BSCs to study viral infection in the brain may reduce the eventual number of animals needed, thereby supporting the 3R’s of animal research (replacement, reduction, refinement). While the number of animals saved by this method is difficult to specifically determine, each postnatal rat pup provides up to five BSCs. As multiple BSCs are obtained from one animal, several replicates, variables, or conditions can be assayed at once. Use of BSCs from littermates can also increase the experimental replicates.

BSCs may also be used for initial testing of therapeutics, reducing the eventual number of animals needed for testing countermeasures. If a therapeutic does not demonstrate efficacy in BSCs, then the need for *in vivo* testing using live animals can be eliminated. Thus, a larger number of potential therapeutics can be screened in BSCs for down-selection prior to *in vivo* testing. BSC experiments, therefore, could serve as a tractable intermediary between high-throughput cell-based antiviral assays and the low-throughput, expensive, and cumbersome BSL-3 animal experiments. While we focused on BSCs obtained from the cortex, BSCs from other regions, such as the cerebellum and brain stem, are possible to address regional differences in permissivity to infection. BSCs from adult animals could also be adapted into this system as a comparator, although they are likely to have a lower viability over time than their postnatal counterparts tested here.

In summary, this study demonstrates that *ex vivo* rat BSCs are permissive to infection by RVFV and that the inflammatory environment induced by infection is similar to *in vivo* studies from adult rats. Since rat BSCs maintain some cellular relationships observed *in vivo*, this model may serve to further understand viral replication kinetics, cellular infectivity, and local antiviral response to RVFV infection in the CNS. Future studies will continue to characterize the impact of RVFV infection in brain tissue and potentially serve as a tool to assess targeted therapeutics.
